# Susceptibility of *Tetranychus urticae* to the Alkaloidal Extract of *Zanthoxylum schreberi* Bark: Phenotypic and Biochemical Insights for Biotechnological Exploitation

**DOI:** 10.3390/biotech13010005

**Published:** 2024-02-20

**Authors:** Ricardo A. Rincón, Daniel Rodríguez, Ericsson Coy-Barrera

**Affiliations:** 1 Biological Control Laboratory, Universidad Militar Nueva Granada, Cajicá 250247, Colombia; ricardo.rinconob@gmail.com; 2 Bioorganic Chemistry Laboratory, Universidad Militar Nueva Granada, Cajicá 250247, Colombia

**Keywords:** two-spotted spider mite, resistance, botanical pesticides, mortality, molecular docking

## Abstract

*Tetranychus urticae* Koch, a phytophagous mite, is one of the most significant crop pests globally. The primary method employed for controlling *T. urticae* involves chemical means, utilizing synthesized products, posing the risk of developing resistance. The urgency for novel strategies integrated into pest management programs to combat this mite is becoming increasingly imperative. Botanical pesticides emerge as a promising tool to forestall arthropod resistance. Among these, extracts from Rutaceae plants, abundant in bioactive specialized metabolites, have demonstrated potential as insecticides and miticides. In this study, various concentrations of alkaloidal extracts sourced from the bark of *Zanthoxylum schreberi* J.F.Gmel. (Rutaceae) were evaluated against *T. urticae* adult females. Furthermore, the extract’s combination with three distinct commercial acaricides (i.e., chlorfenapyr, cyflumetofen, and abamectin) was also assessed for this mite. Chemical characterization of the extract via LC-MS allowed for the annotation of various compounds related to ten benzylisoquinoline-derived alkaloids. The extract, both alone and in combination with commercial insecticides, yielded varying responses, inducing over 40% mortality at 2% *w*/*w*, demonstrating a 90% repellency rate at the same concentration, and exerting a moderate impact on fecundity. These treatments extended beyond phenotypic responses, delving into the biochemical effects on treated *T. urticae* females through an exploration of the impact on four enzymes, i.e., acetylcholinesterase (AChE), glutathione *S*-transferase (GST), esterases (GE), and P450-like monooxygenases (PMO). Employing consensus docking studies and in vitro enzymatic evaluations, it was discovered that the *Z. schreberi*-derived extract and its constituents significantly affected two key enzymes, AChE and GST (IC_50_ < 6 µM), which were associated with the phenotypic observations of *T. urticae* females. The evaluation of alkaloid-rich botanicals showcases promising potential as a relevant biotechnological strategy in addressing mite-related concerns, offering a pathway toward innovative and sustainable pest management solutions.

## 1. Introduction

Amid various strategies for mite control, botanical pesticides or phytochemicals are gaining traction as an alternative to chemically synthesized acaricides [1,2,3]. They serve as a complementary approach to traditional management, exhibiting promising potential in controlling pest arthropods [3]. These pesticides often carry reduced environmental risks and generally have reduced impacts on animal and human health [4]. Within the category of plants displaying pesticide potential, the genus *Zanthoxylum*, a relevant member of the Rutaceae family [5], stands out due to its insecticidal and acaricidal activities [6,7,8]. *Zanthoxylum* is a pantropical genus and comprises ca. 250 species of deciduous and evergreen trees, shrubs, and climbers, being native to warm temperate and subtropical regions across the globe [6,7,8]. Some of the plethora of studies on the pesticide effects of *Zanthoxylum*-derived preparations can be cited. In this regard, *Z. armatum* dichloromethane leaf extract was tested at concentrations of 0.5 and 1% on various pests [9], resulting in mortalities of 46% at 1% for *Helicoverpa armigera*, 42% at 1% for *Plutella xylostella*, 36% and 39% at 0.5 and 1%, respectively, for *Tetranychus urticae*, and 30% and 65% at 0.5 and 1%, respectively, for *Aphis craccivora*. In addition, the insecticidal activity of essential oil from *Z. armatum* and its constituents was evaluated against *Lasioderma serricorne* and *Tribolium castaneum*, exhibiting strong fumigant toxicity on both pests with LD_50_ values of 13.83 and 4.28 mg/L air, respectively [10]. Notably, they identified 1,8-cineole and piperitone as the main compounds responsible for this effect. Furthermore, the high contact toxicity of the essential oil of *Z. schinifolium* against *Sitophilus zeamays* adults was highlighted, with LD_50_ values ranging from 15.93 to 35.31 μg/adult, along with strong fumigant toxicity (LD_50_ from 3.19 to 24.04 mg/L air) [11]. The authors attributed this effect to the compounds sabinene, linalool, and estragole, the primary components quantified through gas chromatography–mass spectrometry (GC-MS). Additionally, extracts of *Z. heitzii* displayed insecticidal activity against *Anopheles gambiae* adults, resulting in mortalities ranging from 97% to 100% when exposed to 1% of Soxhlet and accelerated solvent extraction (ASE) hexane stem bark extracts [12]. A clear toxicity on eggs and females of *Tetranychus viennensis* using extracts from *Z. bungeanum* fruits was also observed, leading to mortalities of 55.57% and 64.14%, respectively, within 24 h post-treatment, with LC_50_ values of 2.09 and 1.13 mg/L [13]. Similarly, the insecticidal effects of the *Z. limonella* extract were demonstrated on individuals of *Culex quinquefasciatus*, resulting in mortality percentages exceeding 95% [14].

Among the world’s problematic pests, the two-spotted spider mite, *T. urticae* Koch, remains a significant threat to numerous crops [1,2]. It is renowned for its rapid resistance development to multiple products [15], including defensive host compounds [16]. Consequently, controlling this pest poses an enduring and challenging task [17]. An essential aspect to consider in understanding the phenomenon of *T. urticae* resistance lies in studies showing the mite’s ability to alter its transcriptional pattern in response to plant defenses, depending on its host plant. A previous study discovered that a susceptible strain of this mite species expresses several genes previously deactivated when transitioning from bean to tomato as a host plant [18]. Moreover, the number of resistance-related genes increases significantly, escalating from the expression of 13 genes after 2 h of changing host plants to 1206 genes after five generations [18]. Therefore, it is crucial to understand the composition of botanical pesticides and how their components affect and/or interact with arthropods such as *T. urticae* [19]. This is a critical fact since, despite promising results in toxicity tests of *Zanthoxylum* extracts on phytophagous arthropods, the mechanisms of action for their components remain mostly unknown, posing challenges in their implementation within crop pest control programs. Consequently, strategies that aid in comprehending how specialized metabolites interact with crop-attacking arthropods become fundamental to further biotechnological exploitation.

Thus, the present study aims to determine the susceptibility of phenotypically diverse rearing system adult females of *Tetranychus urticae* Koch to the alkaloidal extract derived from the bark of *Zanthoxylum schreberi* J.F.Gmel tree, both independently and in combination with commercial acaricides. The evaluation of these extracts was conducted on a mite population previously subjected to multiple pesticide applications, mimicking conditions found in commercial crops and reflecting its variable phenotypic response to several products. Additionally, this study seeks to chemically characterize the test alkaloidal fraction and estimate the biochemical response of *T. urticae* to its detected components through molecular docking and enzymatic activity experiments.

## 2. Materials and Methods

This study was carried out at the Nueva Granada Military University (UMNG) facilities, Campus Nueva Granada (Cajicá-Colombia, 4.94289, −74.01285). This campus is located in a subtropical highland climate (Cfb), based on the Köpen–Geiger climate classification system [20]. The susceptibility tests of *T. urticae* to the plant extract were carried out under laboratory conditions (vide infra). The plant material (i.e., *Z. schreberi* bark) was collected from trees located in the Arbeláez municipality, Cundinamarca on the Fusagasugá-Arbeláez road (4.30170, −74.40593). A voucher specimen was kept at the Herbario Nacional Colombiano under the collection number COL125568.

### 2.1. Mite Rearing

The mite-rearing system for *T. urticae* was established by introducing thirty adult females onto twenty 2-week-old bean plants, with fifteen females placed on each cotyledonary leaf. After 24 h, these females were carefully removed using a fine brush. The plants carrying eggs were then kept in laboratory conditions at 25 ± 2 °C and 60 ± 5% relative humidity for four weeks. Following this, the plants were transferred to the greenhouse and positioned near twenty 6-week-old non-infested bean plants. To ensure a continuous cycle, every 4 weeks, a new set of twenty plants was introduced, allowing the mites to migrate to the fresh material before removing the older plants. This rearing system was divided into two groups: one exposed and one unexposed, each placed in different, isolated greenhouses. In the exposed group, over the 32-week rearing period, mites were intentionally treated with various agrochemical and biological products (outlined in Table 1) typically used for pest and disease control, replicating conditions similar to those in commercial crops. Each agrochemical’s exposure regimen strictly adhered to the manufacturer’s guidelines, utilizing one-fifth of the recommended dosage. It is important to highlight that, in light of the lingering effects of the chemical products employed during mite rearing, fumigations were halted for three days before conducting the susceptibility assays. Meanwhile, the unexposed group was kept for 32 weeks without any chemical treatments.

### 2.2. Preparation of Alkaloidal Extract from Z. schreberi Bark

The branches were separated from collected plants and air-dried in a greenhouse under an average temperature of 22 °C. Once dried, the bark was gently scraped from the branches using a scalpel, weighed, and placed in a container. A 10% HCl solution (2 M) was added to the container, covering the plant material with an additional 20% above, maintaining a pH between 2 and 3. The plant material immersed in the acidic solution underwent agitation in a shaker (90 rpm) for 24 h before filtering to isolate the acidified aqueous phase. After filtration, the plant material underwent another round of treatment with 10% HCl (2 M) to repeat the process iteratively until the highest possible yield of alkaloidal extract was achieved. The filtered aqueous phase underwent alkalinization using 25% ammonium hydroxide (NH_4_OH) until reaching a pH range of 8 to 9. The alkalinized phase was then poured into a separatory funnel, and dichloromethane was added (equal to 30% of the alkalinized phase’s volume). After thorough shaking, the aqueous and organic phases were separated. This process was repeated, ensuring a comprehensive extraction. The obtained organic phase was combined with the initial organic phase in the same container, while the aqueous phase was kept separate. To confirm the presence of alkaloids, Dragendorff’s reagent was used after each extraction cycle over TLC plates, ensuring complete alkaloid removal. The resulting organic phase was concentrated in a round-bottom flask using a rotary evaporator until dichloromethane removal. This extraction process was repeated several times (×8) until 1.32 g of alkaloidal extract was obtained from *Z. schreberi* bark (324 g).

### 2.3. Susceptibility of T. urticae to the Alkaloidal Extract of Z. schreberi

#### 2.3.1. Preliminary Mortality Test with Unexposed Mites

A preliminary trial was conducted to determine the effect of *Z. schreberi* alkaloidal extract on mites for subsequent experiments, following the methodology outlined by Numa et al. (2015) [23], with certain modifications. Briefly, the bioassay comprised a bean leaf with its underside facing upward, which was placed in a petri dish and its edges lined with cotton moistened using distilled water. Ten *T. urticae* females from the unexposed rearing system were situated on each leaf. The bioassay on females was driven by their pivotal role in population dynamics, as they are the primary contributors to new individuals. The net reproductive rate per generation (Ro) or population replacement rate is directly dependent on female reproductive capabilities. Consequently, any lethal or sublethal effects observed in females and oviposition will directly influence population growth. This approach led us to assess the potential of the extracts as a promising strategy for pest control, considering their impact on the crucial factor of population expansion [23]. Subsequently, each dish was sealed with plastic wrap post-application of treatments. The evaluated treatments comprised an absolute control (mites without any application), a positive control (chlorfenapyr at 0.4%), and the alkaloidal extract of *Z. schreberi* at 2%. The application involved locating the *T. urticae* adult females on the bean leaves within the petri dish. Each experimental unit, consisting of the petri dish with the bean leaf, cotton, and mites, received one of the treatments. Each treatment was replicated thrice. The applications were performed using an airbrush in three passes directly on the mites, maintaining a distance of 20 cm. The airbrush was calibrated at 96 drops/cm^2^ and 25 ± 3 psi. Deceased mites were counted 24 h post-application.

#### 2.3.2. Mortality and Repellency Tests for *Z. schreberi* Bark Extract Using Exposed Mites

The experimental setup involved a transparent cylindrical container with a 3.5 cm diameter filter paper disk placed at the bottom, moistened with distilled water. Bean leaf disks, previously immersed in the respective extract solution and air-dried, were then positioned on the paper disk. The container lid underwent modification, featuring a centrally located circular opening. A metal mesh, perforated with small holes, was affixed using silicone around this opening to facilitate ventilation and prevent mites from escaping. Concentrations ranging from 2, 1, 0.5, and 0.25, to 0.125% of the *Z. schreberi* bark alkaloidal extract were evaluated diluted in 10% ethanol. These concentrations were selected since they comprise typical doses for botanical pesticides, and, consequently, they were standardized in previous studies in our laboratory to assess botanical extracts [24]. Additionally, three control treatments were employed: an absolute control (application of distilled water), a relative control (10% ethanol), and a positive control (clorfenapyr at 0.06%). Mortality assessments were conducted at 24, 48, and 72 h post-treatment application. The obtained mortality data were adjusted using Schneider–Orelli’s formula (Equation (1)) [25].

(1) $$Corrected\;Mortality=\frac{b-k}{100-k}\times 100$$

where ‘*b*’ = percentage of individuals killed in the treated experimental unit, ‘*k*’ = the percentage of deceased individuals in the absolute control.

Additionally, repellency measurements were taken at the same time intervals as mortality assessments, recording the number of surviving mites present outside the leaf during the counting process.

#### 2.3.3. Effects on Mortality, Repellency, and Oviposition of *Z. schreberi* Bark Extract Combined with Commercial Acaricides

Using the same methodology described in the previous section, the following treatments were evaluated in this test: absolute control (application of distilled water), relative control (10% ethanol), positive control A (clorfenapyr at 0.06%), positive control B (cyflumetofen at 0.075%), positive control C (abamectin at 0.05%), treatment 1 (0.5% extract), treatment 2 (0.5% extract + 0.06% clorfenapyr), treatment 3 (0.5% extract + 0.075% cyflumetofen), and treatment 4 (0.5% extract + 0.05% abamectin). Mortality assessments were conducted at 24, 48, and 72 h post-treatment application and adjusted using the Schneider–Orelli formula (Equation (1)) [25]. Fecundity was also measured by isolating five surviving females per treatment (after 72 h of exposure to treatments) in petri dishes. The number of eggs oviposited by each female was counted every 24 h over 5 days. Simultaneously with the susceptibility test, the repellent effect of the extract was measured by counting the number of surviving females placed outside the leaves after 24, 48, and 72 h. All laboratory tests were conducted under controlled conditions at 25 ± 2 °C, 60 ± 5% relative humidity, and a 12:12 (light/dark) photoperiod.

### 2.4. LC-MS Characterization of Z. schreberi Bark Extract

The resulting alkaloidal extract was analyzed using liquid chromatography coupled to mass spectrometry (LC-MS) employing Shimadzu LCMS-2020 equipment (Shimadzu Inc., Columbia, MD, USA). Initially, the fraction, dissolved in the mobile phase, was filtered through a 0.2 μm Teflon filter. Separation of the fraction’s components occurred on a high-performance LC (HPLC) system equipped with a standard Premier C-18 column (4.6 mm × 150 mm, 5 μm). This system consisted of a separation module with a photodiode array (PDA) detector, an electrospray ionization (ESI) interface, and a mass detector with a quadrupole analyzer. The flow rate maintained was 0.7 mL/min. Prior tests determined the mobile phases and elution profile to ensure an appropriate resolution and selectivity. The injection volume was set at 5 μL. Compound detection co-occurred at 270 and 330 nm wavelengths, utilizing both the PDA and mass spectrometry detectors. Mass spectra were acquired using ESI in the positive ion mode (scan 100–2000 *m*/*z*). The MS parameters involved a voltage detector at 1.5 kV, a curved desolvation line at 250 °C, a heat block temperature of 400 °C, and a nebulization gas flow of 1.5 L/min. The detection process involved high-resolution MS (HRMS) performed using an Agilent Technologies 1260 Liquid Chromatograph coupled with a quadrupole time-of-flight (Q-ToF) mass analyzer equipped with dual Agilent jet stream electrospray ionization (AJS-ESI) (Agilent, Santa Clara, CA, USA). This LC-HRMS analysis maintained identical chromatographic conditions as previously mentioned. The AJS-ESI ionization functioned in negative ion mode, with specific parameters: capillary voltage (3500 V), drying gas (8 L/min), gas temperature (325 °C), nebulizer pressure (50 psi), sheath gas temperature (350 °C), and sheath gas flow (11 L/min). Meanwhile, the Q-ToF settings included fragmentor voltage (175 V), skimmer voltage (65 V), and octapole radiofrequency peak-to-peak voltage (OCT RF Vpp) (750 V). The compounds were annotated at level 3, based on confidence levels established for communicating metabolite identity through HRMS [26], by combining MS and HRMS data. This annotation process involved a comprehensive diagnostic analysis, considering factors, such as accurate mass, quasimolecular ion, and MS fragments, and supported by phylogeny, chromatographic behavior, and comparison with available literature and KNApSAcK database (http://kanaya.naist.jp/knapsack_jsp/top.html, accessed on 28 December 2023).

### 2.5. Molecular Docking Studies

Upon identification of the detected alkaloids (*n* = 11), their corresponding structures were retrieved from the PubChem database (https://pubchem.ncbi.nlm.nih.gov/, accessed on 28 December 2023) using the simplified molecular input line entry system (SMILES) notation. Avogadro molecule editor (v. 1.2.015) was employed to construct their three-dimensional (3D) structures [27]. Subsequently, these structures underwent energy minimization using the MMFF94 force field and the steepest descent algorithm [27]. On the other hand, three *T. urticae* enzymes, i.e., acetylcholinesterase (AChE), cytochrome P450 monooxygenase (PMO), and glutathione *S*-transferase (GST), were selected for molecular docking studies. However, as crystal structures for these enzymes were unavailable, homology models were constructed. Hence, sequences sourced from the UniProt database [28] were employed to construct these models using the Yasara Structure (v. 19.12.14) [29]. The homology model building macro with default parameters was operated from the respective sequences (i.e., D8V7J9, T1L3S2, and T1K0V7 UniProt entries). The sequences and 3D structures of the modeled enzymes were used as is for molecular docking, adding missing hydrogen atoms and removing ligands, cofactors, and co-crystallized compounds. Co-crystallized inhibitors (i.e., fluorodonepezil, *N*-(4-butyl-2-methylphenyl)-*N*′-hydroxyimidoformamide, and ʟ-γ-glutamyl-*S*-[(9*S*,10*S*)-10-hydroxy-9,10-dihydrophenanthren-9-yl]-ʟ-cysteinylglycine, respectively) of the templates (PDB ID: 7D9O, 6C94, and 6GSV, respectively) were utilized to define the active site and validate the docking calculations (re-docking). Active sites were identified in the specific XYZ coordinates for each enzyme (−12.6, −43.3, 29.7 for AChE, 17.5, −19.2, 29.4 for PMO, and −12.1, −75.2, 126.1 for GST). Molecular docking calculations were initially conducted using the Vina plugin integrated into the molecular graphical system PyMol (v. 1.8) for Microsoft Windows and executed through the molecular graphics laboratory (MGL) tools [30]. Docking simulations were centered on the MMFF-minimized ligand within a cube (24 × 24 × 24 dimensions, 1 Å grid spacing) located at the geometric center of the active sites. Flexible residues (n = 11) within 4 Å of the test ligand were defined. Additional docking simulations for the test alkaloids were performed using Molegro Virtual Docker (MVD) 6.0 [31] and GOLD Suite v5.3 [32], employing different scoring functions under the same docking parameters. The best-docked compounds were ranked using a consensus strategy based on exponential score (ES) calculations, using the reported metrics for exponential consensus ranking (ECR) via Equation (2) [33].

(2) $${ES}_{\left(i\right)}=p\left(r_{i}^{j}\right)=\frac{1}{\sigma }\sum_{j}e^{-\frac{r_{i}^{j}}{\sigma }}$$

where *σ* = exponential distribution (=10), *i* = test compound, *j* = scoring function, and $r_{i}^{j}$ = the ranking per program achieved for each test compound.

Finally, Discovery Studio 2016 Visualizer Client (Biovia, San Diego, CA, USA) [34] was used to visualize the 3D models and 2D residual interaction diagrams of the best poses of top-ranked compounds.

### 2.6. Purification of Selected Alkaloids from Z. schreberi Bark Extract by Semipreparative HPLC

The purification process began with 400 mg of the *Z. schreberi* bark extract, initially subjected to solid-phase extraction (SPE) utilizing Strata^®^ C18-U cartridges (Phenomenex, Torrance, CA, USA) measuring 55 µm, 70 Å, and 500 mg capacity in 6 mL cartridges. Prior to usage, the cartridges were conditioned sequentially with methanol (6 mL) and water (6 mL). After loading the test extract, a water wash (5 mL) followed by elution with methanol (5 mL) isolated the adsorbed components. The obtained eluates, containing the purified extract from the SPE process, underwent semipreparative HPLC isolation. The UFLC Prominence system (Shimadzu Inc., Columbia, MD, USA), operating in semipreparative mode, featured a pump (LC-20AD), a column oven (CTO-20AC), an ultraviolet/visible detector (SPD-20AV), an autosampler (SIL-10AP), and a fraction collector (FRC-10A). Employing a reversed-phase Premier C-18 column (150 × 10 mm, 5 μm), the ten consecutive injections of SPE-purified extract (500 μL per injection, 60 mg/mL in MeOH) were separated at a flow rate of 3 mL/min using solvents A (1% formic acid in H_2_O) and B (1% formic acid in ACN) with an isocratic elution method and monitored at 270 nm. The selected peaks, chosen based on molecular docking, were collected within retention time ranges: 25.5 to 25.9 min (2.6 mg, **3**), 26.2 to 26.8 min (3.8 mg, **4**), 28.3 to 28.7 min (1.5 mg, **5**), 28.8 to 29.9 min (16.7 mg, **6**), and 40.8 to 41.4 min (2.0 mg, **9**), yielding pure compounds. Structural elucidation of these compounds utilized ^1^H and ^13^C NMR spectroscopy, specifically the attached proton test (APT), performed on an Agilent DD2 600 MHz spectrometer (Bruker, Billerica, MA, USA) using CD_3_OD as the solvent. The APT ^13^C NMR data of the isolated compounds aligned with the reported NMR data for berberrubine (**3**) [35], chelerythine (**4**) [36], fagaridine (**5**) [37], berberine (**6**) [38], and zanthoxyline (**9**) [39].

### 2.7. Enzyme Activity and Inhibition

#### 2.7.1. Preparation of the Protein Homogenate from *T. uricae* Adult Females

The protein homogenate from *T. urticae* adult females (n = 50) was prepared by homogenizing them in 500 μL of 0.1 M phosphate buffer (pH 7.2) in an ice bath. After homogenization, the mixture was centrifuged at 12,000× *g* for 20 min at 4 °C. Both untreated mites and those treated with 0.5% *Z. schreberi* bark extract were processed similarly. The protein content within the homogenate was quantified using the linearized Bradford micromethod [40], employing bovine serum albumin as an external standard. A calibration curve was established based on the ratio of absorbance measurements at 590 nm and 450 nm, ensuring linearity with varying protein concentrations [40]. The resulting supernatant was subsequently stored at −20 °C for further enzyme analysis.

#### 2.7.2. Acetylcholinesterase (AChE)

The Ellman method was employed to assess AChE activity in both untreated and *Z. schreberi*-treated mites, following a previous procedure [41]. In brief, protein homogenate (20 µL) was mixed in a microtiter plate well with 1% 100-X triton phosphate buffer (pH 7.8, 145 µL), acetylthiocholine iodide (0.01 M, 25 µL), and 5,5′-dithiobis-(2-nitrobenzoic acid) (0.01 M, 10 µL). This procedure, conducted with three biological and three technical replicates, monitored the enzyme reaction kinetics at 415 nm after a 10 min incubation period using a Varioskan LUX 96-well plate reader (Thermo Fisher Scientific, Waltham, MA, USA). By applying an extinction coefficient of 13.6 mM^−1^cm^−1^ and a path length of 0.3 cm, the kinetic slope was converted to μmoles of product per minute, thereby determining the specific enzyme activity expressed as μmol of substrate/min/mg protein.

#### 2.7.3. Glutathione S-Transferase (GST)

GST activity was assessed using 1-chloro-2, 4-dinitrobenzene (CDNB) as the substrate, following a previous procedure [42]. Briefly, the reaction was initiated by mixing CDNB (50 mM in ethanol, 50 µL) and reduced glutathione (50 mM in 0.1 M phosphate-buffered saline (PBS), pH 6.5, 150 µL) in a 5 mL vial with phosphate buffer (0.1 M, pH 6.5 containing 1 mM EDTA, 2.78 mL). Subsequently, 20 µL of protein homogenate was added, and the solution was thoroughly mixed. An aliquot (200 µL) from the resulting mixture was dispensed into a microtiter plate well for measurement, with the procedure performed in triplicate for both biological and technical replicates. The absorbance of each reaction mixture at 340 nm was then recorded using kinetics on a Varioskan LUX 96-well plate reader (Thermo Fisher Scientific, Waltham, MA, USA) after a 10 min duration. The specific GST activity was calculated using the kinetic slope to convert μmoles of product per minute (9.6 mM^−1^cm^−1^ and 0.3 cm), thereby determining the specific enzyme activity expressed as μmol of substrate/min/mg protein.

#### 2.7.4. Esterase

Enzyme activity of general esterases was assessed using *p*-nitrophenylacetate as substrate, following a previous procedure [43]. In brief, the protein homogenate (10 µL) was mixed in a microtiter plate well with 1 mM *p*-nitrophenyl acetate (200 µL) in 50 mM phosphate buffer pH 7.4. The absorbance was measured at 405 nm after a 10 min incubation period using a Varioskan LUX 96-well plate reader (Thermo Fisher Scientific, Waltham, MA, USA). The specific esterase activity was calculated using the kinetic slope to convert μmoles of product per minute (6.53 mM^−1^cm^−1^ and 0.3 cm), thereby determining the specific enzyme activity expressed as μmol of substrate/min/mg protein.

#### 2.7.5. Cytochrome P450 Monoxygenase (PMO)

The PMO activity was measured by reducing hydrogen peroxide to oxidize 3,3′,5,5′-tetramethylbenzidine (TMBZ), following a previous procedure [44]. Briefly, the protein homogenate (20 µL) was mixed in a microtiter plate well with 6 mM TMBZ (200 µL) working solution (TMBZ (0.01 g) in methanol (5 mL) and 0.25 M sodium acetate (pH 5.0, 15 mL)) and 3% H_2_O_2_ (25 µL) for 30 min at 25 °C. After this incubation period, the absorbance was read at 630 nm on a Varioskan LUX 96-well plate reader (Thermo Fisher Scientific, Waltham, MA, USA). The standard curve of heme peroxidase activity was prepared using Cytochrome C from equine heart (Merck, Milwakee, WI, USA). Total Cytochrome P450 monoxygenase was expressed as µmoles of Cytochrome P450 equivalent units (CPEU)/min/mg protein.

#### 2.7.6. Enzyme Inhibition

Further investigation involved determining the inhibition of the test enzymes (i.e., AChE, GST, esterase, and PMO). Hence, various concentrations (ranging from 0.01 to 1000 µg/mL and 0.03 to 300 µM) of alkaloidal extract and purified alkaloids, respectively, were employed under the same procedures for assessing enzyme activity mentioned above. The final absorbance measurements of each treatment (*At*) and control (*Ac*) were related to the calculated enzyme inhibition percentage using Equation (3).

(3) $$Enzyme\;Inhibition\;(\%)=\frac{Ac-At}{At}\;*\;100$$

This determination was performed for each treatment concentration (extract and purified compound) to establish dose–response curves. The IC_50_ (half-maximal inhibitory concentration in μg/mL for extract and μM for pure compounds) was calculated via nonlinear regression analysis using GraphPad 7.0 (GraphPad Software LLC, Boston, MA, USA).

### 2.8. Experimental Design and Statistical Analysis

The experimental design employed for bioassays using extract and acaricides followed a completely randomized design (CRD). Data analysis utilized the generalized linear models (GLMs) with the statistical software R 4.3.1 [45]. A binomial distribution was assumed for the analyses of mortality and repellency, while a Poisson distribution was used for oviposition analysis. The enzyme activity and inhibition data underwent analysis of variance (ANOVA) to identify significant differences among them. Normal data distribution was checked for each analysis using the Shapiro–Wilk normality test. Subsequently, a post hoc multiple comparison Tukey test was applied whenever the ANOVA revealed significant differences.

## 3. Results

### 3.1. Preliminary Test Using Unexposed Mite Population

**Figure 1:**
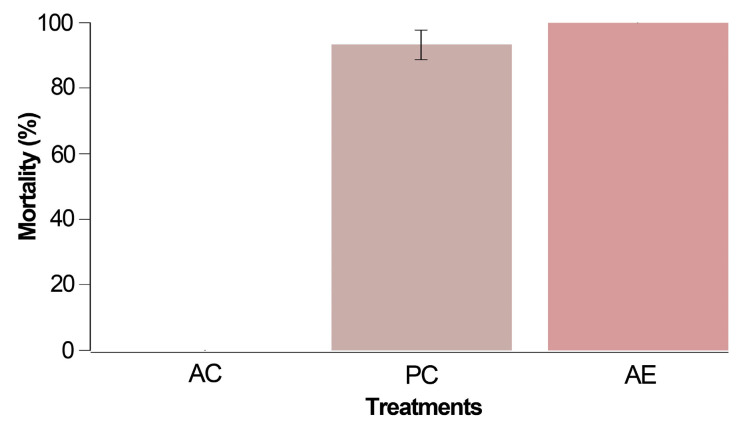
Corrected mortality after 24 h for the preliminary trial carried out with three treatments: AC (absolute control, i.e., *T. urticae* females without application), PC (positive control using 0.4% chlorfenapyr), and AE (2% *Z. schreberi* alkaloidal extract). The data are expressed as a percentage ± standard error.

No mortality was noted in the absolute control group in this preliminary test involving mites unexposed to chemical products during their rearing phase. However, within 24 h of conducting this bioassay (Figure 1), both the positive control (PC) and treatment with alkaloidal extract (AE) exhibited mortality rates of 93.3% and 100%, respectively. Notably, the mortality recorded with the *Z. schreberi* extract at 2% surpassed that of the positive control (chlorfenapyr) when assessing unexposed *T. urticae* females.

### 3.2. Mortality and Repellency Test for Exposed Mite Population

**Figure 2:**
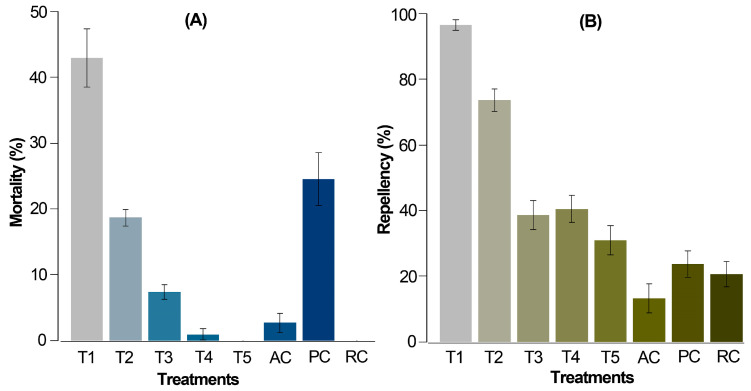
Corrected average mortality (**A**) and repellency (**B**) of *T. urticae* females after 72 h from application by the leaf-immersion method (6 repetitions per treatment and 20 mites per repetition). T1 = 2% extract, T2 = 1% extract, T3 = 0.5% extract, T4 = 0.25% extract, T5 = 0.125% extract, PC = 0.06% chlorfenapyr (commercially recommended dose), RC = relative control, 10% ethanol, AC = absolute control, distilled water. The data are expressed as percentage ± standard error.

For the chemical-product-exposed mite population, the accumulated mortality and repellency-only outcomes after 72 h (Figure 2A) involved lower mortality rates in comparison to the preliminary test.

In this case, the alkaloidal extract at 2% (T1) and chlorfenapyr (PC) resulted in significantly higher mortalities compared to the relative and absolute controls, registering values of 43.0% and 24.6%, respectively. A dose-dependent response was evident as the concentration of *Z. schreberi* extract decreased from 2 to 0.125% (T1 to T5). At 0.125% (T5), no mortality was observed in *T. urticae* females. Remarkably, T1 induced approximately twice the mortality compared to the positive control (PC), despite PC being administered at a lower dose (commercially recommended). The application of water (AC) and 10% ethanol (RC) induced the lowest mortality (ca. 3%) and no mortality, respectively.

Regarding repellency, T1 and T2 exhibited significantly higher repellent effects (94.5 and 73.4%, respectively) compared to the controls AC and RC (12.3 and 19.8%, respectively) (Figure 2B). Extract doses at 0.5, 0.25, and 0.125% (T3 to T5) demonstrated a moderate repellent effect on *T. urticae* adult females (<40%). As anticipated, treatment TP (0.06% chlorfenapyr) showed the lowest repellency, as it induces mortality rather than repellent action in individuals.

### 3.3. Mortality, Repellency, and Oviposition Test for Extract Combined with Acaricides Using the Exposed Mite Population

**Figure 3:**
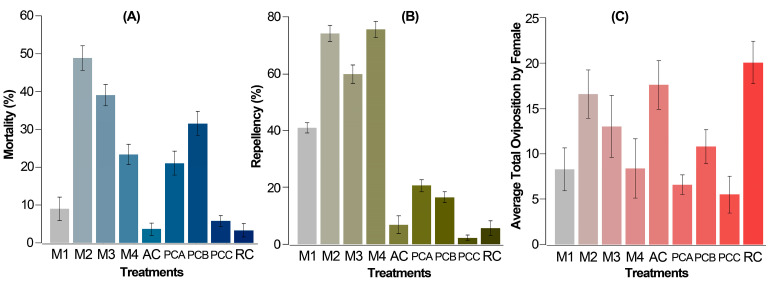
Corrected average mortality (**A**), repellency (**B**), and fecundity (**C**) of *T. urticae* females. Mortality (**A**) and repellency (**B**) after 72 h from the application by the leaf-immersion method for the two replicates (each replicate of 6 repetitions per treatment and 20 mites per repetition). Average fecundity (**C**) per *T. urticae* female per day after 120 h from the application by the leaf-immersion method for the two replicates (5 repetitions per treatment, each involving one female). M1 = 0.5% extract, M2 = 0.5% extract + 0.06% chlorfenapyr, M3 = 0.5% extract + 0.075% cyflumetofen, and M4: 0.5% extract + 0.05% abamectin, AC = absolute control, distilled water, RC = relative control, 10% ethanol, PCA = 0.06% chlorfenapyr, PCB = 0.075% cyflumetofen, PCC = 0.05% abamectin. Acaricides were used at the commercially recommended dose. The data are expressed as a percentage ± standard error.

The mortality rate after 72 h with 0.5% *Z. schreberi* extract (M1) stood at 8%, notably lower when compared to the combinations of extract with acaricides and the acaricide used alone (above 20%), except for 0.05% abamectin (PCC) (ca. 6%), which did not exhibit significant differences in comparison to the absolute and relative controls (AC and RC) (Figure 3A). However, a substantial increase in mortality (until 25.7%) was observed in M4 (0.5% extract + 0.05% abamectin), suggesting a positive influence of the plant extract on the combined toxic effect. This pattern was consistent across other acaricides (PCA versus. M2 and PCB versus. M3), where their combined effect with the alkaloidal extract (M2 and M3) yielded higher mortalities compared to the acaricide alone (PCA and PCB) at the same recommended concentration. Particularly, the combined effect with chlorfenapyr exhibited an approximately 2.5-fold increase in its additive effect.

On the other hand, after 72 h, all combined treatments (M2 to M4) and two acaricides (PCA and PCB) exhibited significantly higher repellency compared to the absolute control, except for PCC. Notably, the most pronounced effect was witnessed in all combined treatments involving the *Z. schreberi* bark extract and acaricide, showcasing repellency values surpassing 60% (Figure 3B). This stood in contrast to the outcomes from the bioassay solely with the plant extract (around 40%) and the individual acaricides used alone (below 40%). The best repellent effect was observed for extract combinations with chlorfenapyr and abamectin (above 75%).

Finally, the average fecundity per *T. urticae* female over 5 days showed a discernible pattern, notwithstanding a relatively high standard deviation. This variation is typical and can be attributed to the population dynamics of a diverse female population, since the replacement rate is directly contingent upon the reproductive capabilities of females. Hence, average fecundity indicates a statistically significant reduction in PCA (chlorfenapyr 0.06%), PCB (0.075% cyflumetofen), PCC (0.05% abamectin), M1 (0.5% extract), and M4 (0.5% extract + 0.05% abamectin) compared to the controls AC and RC. However, treatments M2 and M3 did not exhibit significant differences from any of the controls (Figure 3C), suggesting no notable impact on fecundity from extract combinations, except M4.

### 3.4. Chemical Characterization of Z. schreberi Bark Extract

**Figure 4:**
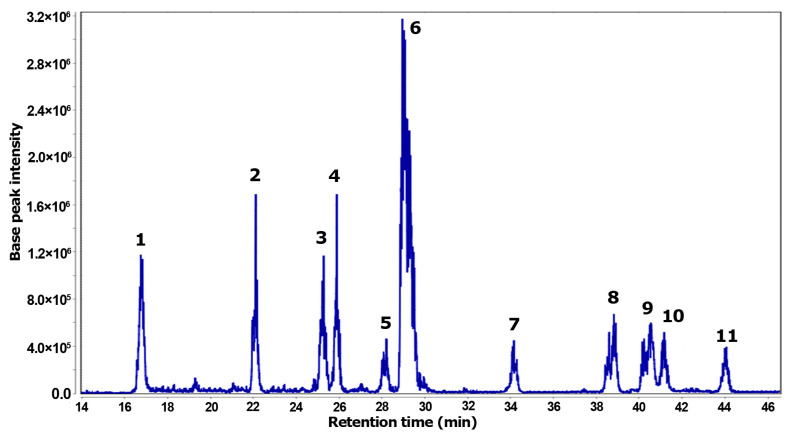
Total ion chromatogram (TIC) obtained by LC-MS from the alkaloidal extract of *Z. schreberi*.

The total ion chromatogram (TIC) represents the resulting analysis of the *Z. schreberi* alkaloidal extract using LC-ESI-MS (Figure 4). This analysis revealed eleven distinctive MS features, each corresponding to individual detected metabolites. Two zones in the TIC were evidenced, comprising polar (14–30 min) and less polar (33–45 min) compounds. Among the compounds detected by MS, the most abundant compound was **6**, while less abundant compounds were **5**, **7**, and **1**.

Through a comprehensive diagnostic analysis of both chromatographic and MS data, metabolites **1**–**11** were annotated and are listed in Table 2, with accurate mass errors below |5| ppm. Among the annotated compounds, ten were related to benzylisoquinoline-like alkaloids, encompassing five protoberberines (**1**–**3**, **6**, **8**) and five benzophenanthridine (**4**–**5**, **9**–**11**) alkaloids. Additionally, a quinoline alkaloid (**7**) was also one of the detected compounds in the bark extract. Compounds **1**–**6** correspond to more polar alkaloids, characterized by quaternary ammonium and phenolic moieties, while compounds **7**–**11** represent less polar alkaloids.

### 3.5. Enzyme Activities of Z. schreberi-Treated and Untreated Mites (Exposed Population)

The examination of *T. urticae* adult females from the exposed mite population delved into exploring the activity of key enzymes recognized for their role in biochemical defense against xenobiotics, especially for detoxification purposes [15]. Consequently, four enzymes—acetylcholinesterase (AChE), glutathione *S*-transferase (GST), P450 monooxygenase (PMO), and general esterases—were explicitly chosen to investigate this biochemical response in both 0.5% *Z. schreberi*-treated and untreated mites (Table 3). After the 72 h treatment application, the general esterase activity exhibited no significant changes between treated and untreated mites (ca. 440 µmoles of substrate/min/mg protein). However, PMO displayed an approximately three-fold increase in activity in mites treated with the extract. Remarkably, AChE and GST activities were notably reduced (2- and 9-fold, respectively) in treated mites compared to their untreated counterparts.

### 3.6. Molecular Docking Studies

Considering the previous effect regarding the enzyme activity in protein homogenates from untreated and extract-treated mites, we aimed to computationally explore the capacity of benzylisoquinoline alkaloids annotated in the *Z. schreberi* bark extract to interact with the active site of three enzymes (i.e., AChE, GST and PMO) by performing molecular docking simulations. Consequently, homology model structures of test enzymes were obtained from the respective *T. urticae* enzyme sequences (vide supra), involving good-quality parameters (Z-scores between 0.3 and 0.6). Thus, the resulting structures (i.e., for *Tu*AChE, *Tu*GST, and *Tu*PMO) served as templates for preparing test enzyme structures for docking studies [30]. The co-crystallized ligands were re-docked for validation, showing good convergence with RMSD of atomic positions < 0.6 Å compared to the original structures.

Subsequently, an extended structure-based virtual screening was performed on the eleven annotated alkaloids (**1**–**11**). The compounds were docked using Autodock/Vina with flexible residues, and two additional search algorithms were employed to ensure a consensus docking strategy (i.e., MVD and GOLD). The resulting scores from the three docking programs were used to rank the compounds via exponential consensus ranking (ECR) [33] (Table 4). This metric revealed that co-crystallized inhibitors (**12**–**14**) exhibited the most favorable consensus ranking (ES > 0.23), with berberine (**6**) emerging as the best-docked alkaloid for *Tu*AChE and *Tu*GST (ES > 0.21). Meanwhile, γ-fagarine exhibited the best ranking for *Tu*PMO (ES > 0.25), although it fell below the respective co-crystallized inhibitor (**14**). In the case of AChE, compounds chelerithryne (**4**) and zanthoxyline (**9**) secured the third and fourth positions, respectively (ES > 0.2), whereas *N*-methylcanadine (**2**) and berberrubine (**3**) (ES > 0.2) also attained identical rankings for *Tu*GST. Thus, protoberberine alkaloids featuring a 2,3-methylenedioxy group exhibited a strong docking performance for *Tu*GST, while protoberberine/benzophenanthridine alkaloids having an aromatized ring B displayed better performance for *Tu*AChE. Contrarily, molecular docking with *Tu*PMO demonstrated a consensus ranking with significantly lower scores for the test alkaloids compared to the inhibitor.

#### Binding Mode of Top-Ranked Compounds within Active Sites of TuAChE and TuGST

**Figure 5:**
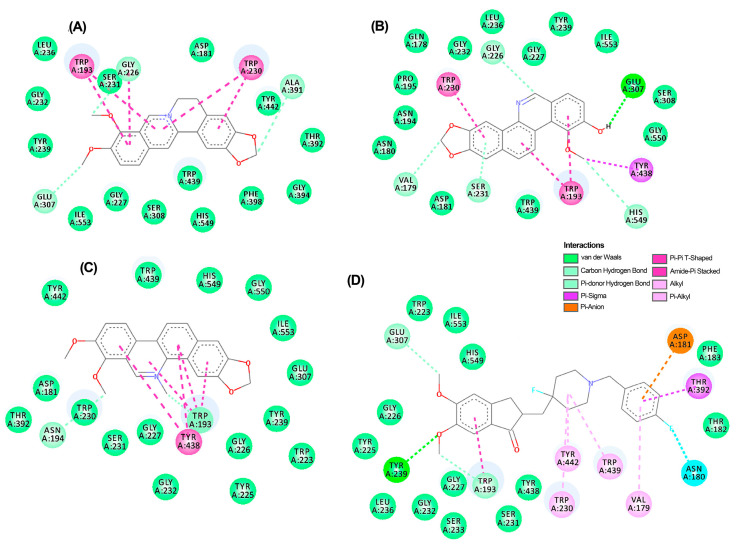
Two-dimensional residual interaction diagrams between *T. urticae* acetylcholinesterase (*Tu*AChE) and the compounds berberine **6** (**A**), zanthoxyline **9** (**B**), chelerythrine **4** (**C**), and inhibitor **12** (**D**). Dash lines between the ligand and amino acids represent interactions, and their color corresponds to the type according to the interaction panel in the right-middle of this Figure.

**Figure 6:**
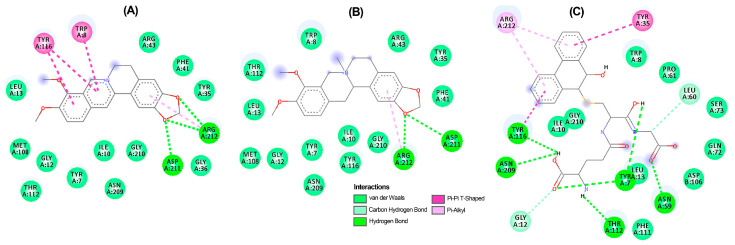
Two-dimensional residual interaction diagrams between *T. urticae* glutathione *S*-transferase (*Tu*GST) and the compounds berberine **6** (**A**), *N*-methylcanadine **2** (**B**), and inhibitor **13** (**C**). Dash lines between the ligand and amino acids represent interactions, and their color corresponds to the type according to the interaction panel in the bottom of this Figure.

A thorough analysis of the binding mode of the top-ranked compounds for each enzyme was conducted using 2D and 3D interaction diagrams. Specifically, *Tu*AChE and *Tu*GST showed promising interactions with test alkaloids, explaining the outcome of the consensus docking. Berberine (**6**) is positioned exceptionally well within the active sites of both enzymes (Figure 5A and Figure 6A), exhibiting notable hydrogen bonds and hydrophobic pi–pi interactions. Within the *Tu*AChE···**6** complex, critical contacts occurred between the aromatic rings of **6** and TRP193 and TRP230 residues via pi–pi interactions (Figure 5A). 

In the *Tu*GST···**6** complex, hydrogen bonds were formed between methylenedioxy oxygens and ASP211 and ARG2012 residues, along with a pi–pi interaction involving TYR116 and TRP8 residues. These interactions suggest stability within the simulated complexes, potentially elucidating the compound’s promising acaricidal effect. These findings emphasize the potential of berberine-like alkaloids as acaricidal agents, urging further investigation. The related benzophenanthridine alkaloids, particularly **9**, displayed similar pi–pi interactions to **6** (Figure 5B), although the best pose exhibited a differing orientation within *Tu*AChE’s active site. Meanwhile, alkaloid **4** and inhibitor **12** demonstrated distinct binding patterns (Figure 5C,D). Conversely, protoberberine alkaloid **2** lacked an aromatized ring B and pi–pi interactions within *Tu*GST’s active site (Figure 6B) but displayed similar hydrogen bonds to **6**. In contrast, inhibitor **13** presented a different binding mode compared to the top-ranked alkaloids for *Tu*GST (Figure 6C).

**Figure 7:**
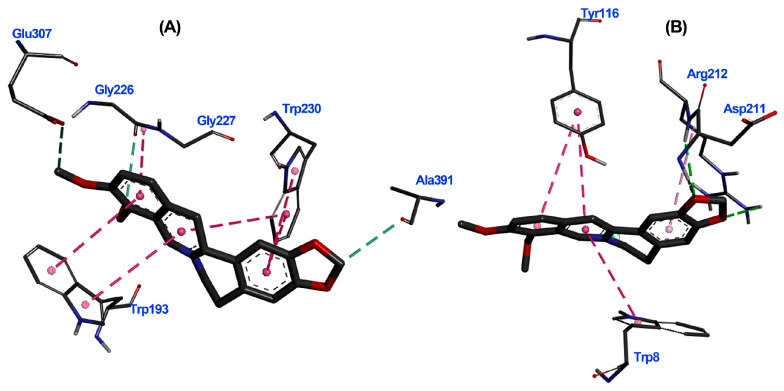
Three-dimensional interaction diagrams of berberine (**6**) within the active site of *T. urticae* acetylcholinesterase (*Tu*AChE) (**A**) and *T. urticae* glutathione *S*-transferase (*Tu*GST) (**B**). Dots represent the Pi/alkyl interaction centroid. Interacting amino acids in thin grey sticks and ligand in thick dark grey sticks. Dash lines between the ligand and amino acids represent interactions, and their color corresponds to the interaction type (Green: Hydrogen bond; Pink: Pi-Pi T-shaped; Light pink: Pi-alkyl).

The 3D interaction diagrams for *Tu*AChE···**6** and *Tu*GST···**6** complexes revealed interaction distributions (Figure 7A,B), particularly highlighting the pi–pi interaction with aromatic residues like TRP193 and TRP230 for *Tu*AChE and TRP8 and TYR116 for *Tu*GST. The planar nature of alkaloid **6** favored these interactions.

### 3.7. Enzyme Inhibition of Z. schreberi Extract and Isolated Alkaloids

**Figure 8:**
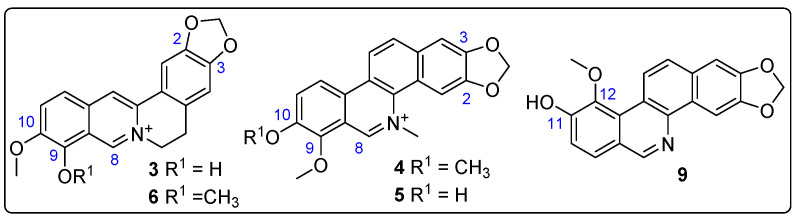
Structures of isolated alkaloids from *Z. schreberi* bark extract. Blue numbers represent carbon numbering of alkaloid moiety.

The structure-based virtual screening identified various alkaloids (e.g., **3**, **4**, **6**, **9**) as key metabolites significantly influencing the interaction with test enzymes, specifically AChE and GST. To validate this recognition of potential bioactives, five alkaloids underwent purification by semipreparative HPLC separation from *Z. schreberi* bark extract (vide supra). Their structures were then elucidated using nuclear magnetic resonance (NMR). This elucidation confirmed the annotation of these compounds (i.e., **3**–**6**, **9**), identified as known metabolites, as their ^13^C NMR profiles matched those reported for berberrubine (**3**) [35], chelerythine (**4**) [36], fagaridine (**5**) [37], berberine (**6**) [38], and zanthoxyline (**9**) [39], respectively. The structures of these isolated and identified compounds are presented in Figure 8.

The inhibitory potential of the alkaloidal extract and isolated compounds **3**–**6** and **9** was assessed against test enzymes in the protein homogenate of *T. urticae*. Table 5 displays the IC_50_ values for these substances.

*Z. schreberi* bark extract and the isolated alkaloids showed poor inhibition against PMO and esterase enzymes, except for alkaloid **9**, demonstrating a moderate PMO inhibition (IC_50_ = 8.37 μM). Conversely, alkaloids generally demonstrated moderate-to-good activity against GST and AChE. The extract displayed moderate inhibition (IC_50_ > 8.5 µg/mL), while berberine (**6**) exhibited the most potent inhibitory effect against GST and AChE (IC_50_ = 1.24 and 0.325 μM, respectively). Noteworthy is berberrubine (**3**), which displayed the second-best activity against GST (IC_50_ = 5.61 μM), while chelerythrine (**4**) exhibited the second-best inhibition against AChE (IC_50_ = 1.86 μM). Compound 5 showed the lowest activity against both GST and AChE enzymes (IC_50_ = 112 and 14.6 μM, respectively), which agrees with the structure-based virtual screening through consensus ranking.

## 4. Discussion

Most studies have been conducted on mite populations susceptible to toxic products, be they synthetic or botanical. In some cases, evaluations have been carried out on populations showing specific resistance to a certain compound [15]. Nevertheless, the activity of pesticides is generally not evaluated on populations exposed to repeated applications of multiple pesticide products, as typically carried out by farmers in commercial crop conditions. Consequently, the effectiveness of these evaluated components against populations susceptible or resistant to individual compounds might be limited when implemented in field conditions. In our study, the mortality observed with the 0.05% concentration of the *Z. schreberi* extract surpassed that of the positive treatment (chlorfenapyr) when evaluating *T. urticae* females unexposed to the products listed in Table 1. This outcome underscores the high susceptibility of individuals not previously exposed to agrochemicals, evident in their response to both the extract and chlorfenapyr. However, this interpretation warrants caution since the notably high mortality seen with chlorfenapyr is largely due to its concentration (0.4%), nearly seven-times higher than the commercial dosage (0.06%), a factor crucial to consider (Table 1). However, *Z. schreberi* extract exhibited potential against a susceptible mite population, which agrees with previous studies. It was previously reported that mortality rates were between 35% and 40% for *T. urticae* when exposed to extracts derived from *Z. armatum* at concentrations of 0.5 and 1% [9]. However, these concentrations failed to demonstrate differences compared to the absolute control in our bioassay. This could potentially be attributed to the mite’s ability to develop resistance against various compounds. It is worth noting that the mortality recorded for chlorfenapyr at its commercial dosage was merely 24.6%. As a reference point, a previous study considered mite groups with a survival range between 30% and 50% after 72 h of exposure to concentrations from 120 to 1920 mg/L as originating from resistant populations [46]. Hence, the prevalence of a substantial proportion of resistant individuals within the rearing population exposed to the diverse products listed in Table 1 cannot be discounted. This species frequently develops resistance, mainly attributed to the array of induced detoxifying proteins, such as P450 monooxygenases, esterases, and glutathione *S*-transferases [47]. Earlier, the mite resistance to the compound abamectin was associated with the action of cytochrome P450 monooxygenase enzymes [48]. Their research also indicated a proportional increase in glutathione *S*-transferase enzyme activity concerning the mite’s resistance factor to abamectin. A similar investigation established a correlation between elevated levels of oxidases and esterases and resistance to the active ingredient fenpyroximate in *T. urticae* [49]. While no genetic analysis was conducted in our study to assert the definitive influence of detoxifying enzymes on the mite’s resistance to different treatments, the conditions under which the rearing was established suggest the involvement of these enzymes in the low mortalities observed in the conducted tests.

The mortality percentage recorded at a concentration of 2% aligns with previous observations using essential oils of *Citrus sinensis* Osbeck (Rutaceae) and *Citrus limon* (L.) Burm. F. (Rutaceae), inducing mortalities of 45.6% and 34.9% in *T. urticae* adults [50]. A mortality of 55% in adults was also reported when using the essential oil of *Citrus aurantium* L. [51], albeit at a concentration of 0.1%, significantly lower than the 2% concentration in our study. Moreover, our results coincide with other studies encompassing plant families beyond Rutaceae. For instance, a 50% mortality in *T. urticae* was demonstrated with concentrations of 30 ppm and 80 ppm of two components from *Calceolaria andina* Benth extract (Solanaceae) [52], and a 50% mortality of this mite was recorded utilizing a 1% concentration of *Quassia* sp. leaf extract (Simarubaceae) [53]. In comparison, a mortality of 45% in *T. urticae* adult females was determined using the *Capsicum annuum* L. (Solanaceae) fruit extract [54]. Similar mortalities were achieved when assessing the toxic effect of the extract from *Waltheria indica* L. (Sterculiaceae) and *Amaranthus viridis* L. (Amaranthaceae), reporting mortalities between 40% and 60% at concentrations ranging from 0.25% to 0.5% [55]. Extracts from various plants of the Umbelliferae family were tested and observed mortalities between 20% and 50% at concentrations of 1% [56].

In addition, while limited, studies exploring the repellent effect of crude, non-volatile extracts from Rutaceae family species that examine essential oils are available. Hence, our findings align with those previously reported on observing repellent action in *T. urticae* females when testing essential oil from *Citrus sinensis* Osbeck (Rutaceae) fruits at a concentration of 1% [57]. They attributed this effect to all identified compounds present in the essential oil. Similarly, reports of repellent activity in *T. urticae* span beyond Rutaceae to other plant families. For instance, repellent activity in concentrations ranging between 1.56 and 50 mg/L of azadirachtin A (from *Azadirachta indica* A. Juss., Meliaceae) against *T. urticae* adult females was evidenced [58]. Additionally, a repellent effect in the fruit extract of three Solanaceae species within the *Capsicum* genus was observed when assessed on *T. urticae* adult females [54].

Regarding the commercial acaricides used alone, clorfenapyr promoted a mortality rate in *T. urticae* after 72 h of 20.9%. This value contrasts with former studies, which reported LD_50_ values of 0.08% and 0.29% for two resistant strains of *T. urticae* to the same active ingredient [59]. This discrepancy suggests potential resistance against clorfenapyr in the mite population used in this study. Similarly, concerning the cyflumetofen at 0.075%, a mortality rate of 32.5% was observed in *T. urticae* adult females. An LD_50_ of 0.0016% was reported for a resistant strain of *T. cinnabarinus* Boisdu-Val to cyflumetofen [60], while an LD_50_ of 0.00011% was estimated for a susceptible strain of *T. urticae* [61]. These substantially lower concentrations compared to those used in this study suggest potential resistance to cyflumetofen in the mites of the test population. Finally, the abamectin at a commercial dose of 0.05% did not show significant differences from the absolute control, indicating a minimal impact on the mortality of *T. urticae* females from the test population. This suggests potential resistance to abamectin in the mite population used in this study. Additionally, cyflumetofen induced the highest mortality among the tested acaricides, combining this miticide and the *Z. schreberi* extract (M3) with slightly improved mortality rates (Figure 3). This suggests that the extract has a moderate additive effect with cyflumetofen. However, contrasting results were observed for the chlorfenapyr treatments since, if the acaricide was used alone, the mortality was significantly lower compared to the acaricide combined with the *Z. schreberi* bark extract. A similar trend was observed for abamectin combined with the bark extract. These facts suggest a plausible synergistic or additive toxic effect on *T. urticae* for these acaricides in combination with *Z. schreberi* bark extract as a botanical pesticide.

Although a biopesticide has a good cost–effectiveness ratio, its production and effective usage necessitate adherence to specific requirements to ensure efficiency, quality, and environmental safety [62]. In addition, notwithstanding their potential benefits, biopesticides have yet to attain the desired level of adoption needed to replace the prevalent use of commercially available synthetic pesticides, constituting a modest fraction of the global crop protection market, comprising just 5% (ca. USD 3 billion) [63]. This fact is primarily a result of the delayed development and commercialization of new biopesticide products, impeding their widespread acceptance. Additionally, among the pros and cons, first and foremost, a reliable source of high-quality raw materials, such as bioactive plant extract with potent pesticidal properties, is essential. In this regard, the main limitation of the direct use of *Z. schreberi* bark extract as a botanical pesticide is related to its production due to the limitations for its cultivation to source its bioactive agents (i.e., benzylisoquinoline-like alkaloids), which demand controlled environments and optimized growth conditions of a tree to maximize their yield and efficacy [64]. Furthermore, botanical pesticide production from a tree with restricted distribution is a crucial limitation since *Z. schreberi* extends from western and southern Mexico to Venezuela, Bolivia, and the Caribbean, predominantly thriving in the wet tropical biome [65].

On the other hand, the production facilities must adhere to stringent quality control measures throughout the process, from cultivation and extraction to formulation. This production includes rigorous testing for purity, potency, and absence of contaminants to meet regulatory standards in established laboratories [66]. To minimize the environmental impact, sustainable and eco-friendly practices should be integrated into the production process. Additionally, a thorough understanding of the target pest and its biology is crucial for designing biopesticides that are not only effective but also selective, minimizing non-target impacts. Hence, successful biopesticide production requires a harmonious integration of scientific knowledge, equipment, labor, technological expertise, and a commitment to environmental stewardship [62,63,64].

In this context, identifying active principles within a bioactive extract can launch a further possibility of finding hits, which can be further explored. This alternative would be pursued with a chemical characterization of the botanical pesticide, e.g., through LC-MS. Thus, the annotated compounds by LC-MS agree with prior reports [67], confirming the presence of benzylisoquinoline alkaloids, which are recognized as chemotaxonomic markers within the genus [68]. Berberine, a well-studied alkaloid and a representative of the protoberberine alkaloid family, emerges prominently within the identified compounds [69,70]. Its wide-ranging biological activities include leishmanicidal and antimicrobial effects, and it is recognized for imparting yellow coloration to certain plant species within this genus [70]. Due to the presence of this type of biologically active specialized metabolite and the phenotypic response related to mortality, repellency, and fecundity reduction in the *Z. schreberi* extract, and the fact that the enzyme activity in protein homogenate of extract-treated *T. urticae* adult females was reduced for AChE and GST, a deeper exploration to examine their action on these enzymes and a plausible role on mite resistance was conducted. Thus, molecular docking, chosen as the primary method for structure-based bioactive discrimination, simulates the binding of low-molecular-weight compounds within target enzyme active sites, predicting potential binders and non-binders [71]. The consensus docking analysis revealed that particular structural features of protoberberine- and benzophenanthridine-type alkaloids are crucial for enzyme interaction. Therefore, protoberberine alkaloids (e.g., **2**, **3**, **6**), characterized by a 2,3-methylenedioxy group, demonstrated robust docking efficiency concerning *Tu*GST, whereas protoberberine/benzophenanthridine alkaloids with an aromatized ring C (e.g., **3**–**6**, **9**, **11**) showcased superior performance concerning *Tu*AChE, as observed in the 2D and 3D interaction diagrams. In this regard, the top-ranked alkaloids may serve as viable candidates for *Tu*AChE and *Tu*GST inhibition. Conversely, alkaloids in the extract prompt a consideration of their potential interactions with *Tu*PMO, suggesting their likely unsuitability as candidates for inhibiting this *T. urticae* enzyme. This computational insight offers a plausible explanation for variations in enzyme activity observed within protein homogenates of untreated and *Z. schreberi* extract-treated *T. urticae* adult females. Indeed, the experimental confirmation of enzyme inhibition revealed that berberine (**6**), berberrubine (**3**), and chelerythrine (**4**) are the active constituents present in the *Z. schreberi* extract. In a recent study, a collection of 34 isoquinoline alkaloids exhibited activity against both acetylcholinesterase (AChE) and butyrylcholinesterase (BuChE) from various sources. The findings revealed that berberine (**6**) emerged as the most potent alkaloid among the tested compounds (IC_50_= 0.72 μg/mL) [72], which coincide with the present outcome against *Tu*AChE. Additionally, it appeared that a 9,10-dimethoxylated aromatic moiety on protoberberine and benzophenanthridine alkaloids had a more favorable effect on enzyme inhibition for both *Tu*AChE and *Tu*GST compared to 9-hydroxy,10-methoxy-bearing compounds. This observation is supported by the superior inhibition displayed by compounds **6** and **4** (having dimethoxyl moiety) in comparison to **3** and **5** (having hydroxymethoxyl moiety), aligning with the findings from molecular docking results. These compounds had not been previously evaluated for acaricidal activity against *T. urticae* populations, making this study the first to explore their impact on the phenotypic and biochemical response of this pest mite. In this sense, these identified bioactive compounds are likely accountable for the observed effects of the extract, whether used alone or in combination with acaricides. Their role in potentially mitigating resistance could significantly influence integrated pest management strategies. Future investigations will explore the impact of isolated compounds on the phenotypic and molecular response of both susceptible and resistant mite populations. The limited quantity of isolated compounds in this study prevented such comprehensive assessments, prompting the need for further research in this direction. In summary, exploring benzylisoquinoline alkaloids as acaricides against *T. urticae* presents a promising alternative in pest management strategies. Their diverse properties, potential modes of action, and structural variability offer intriguing prospects for effective and environmentally sustainable control measures against this economically significant pest.

## 5. Concluding Remarks

This study involving the alkaloidal extract of *Z. schreberi* revealed significant effects on *T. urticae* adult females when combined with commercial acaricides and when used separately. At a concentration of 2% *w*/*w*, it induced mortality rates exceeding 40%, exhibited a repellency rate of 90%, and exerted a moderate influence on fecundity. These findings underscore the potential of this extract as a means of controlling these mites. Indeed, a compelling discovery was the noticeable repellent impact of the bark extract on *T. urticae* adult females, both in tandem with chemically synthesized commercial acaricides and independently. This repellent effect hints at the extract’s promise in pest management strategies. However, interestingly, the alkaloidal fraction of the bark extract did not significantly affect the fecundity of *T. urticae* adult females, suggesting a lack of sublethal effects associated with the extract’s compounds. Among the eleven annotated alkaloids in the bark extract, the majority belonged to the benzylisoquinoline group, characteristic of the Rutaceae family, with berberine being the predominant compound, which exhibited good performance in inhibiting AChE and GST enzymes (IC_50_ < 21 µM). Overall, these findings shed light on the complex interactions between the alkaloidal extract of *Z. schreberi* and *T. urticae*, pointing toward potential mechanisms of defense and resistance that merit deeper exploration, highlighting the benzylisoquinoline-like hits (alkaloids **3**, **4**, **6**, **9**) as enzyme inhibitors to examine broader applicability in further studies.

## Figures and Tables

**Table 1:** Agrochemicals and biological products used in the rearing system for exposing *T. urticae* females prior to bioassays.

Commercial Product	Effect	Active Ingredients	Mechanism of Action ^a^
Quorum	Fungicide	chlorothalonil/dimethomorph	Lipid biosynthesis inhibition
Connect Duo	Insecticide	imidacloprid/β-cyfluthrin	Competitive modulator of acetylcholine receptors/sodium channel modulator
Abamectin	Acaricide	abamectin	Allosteric modulator of chlorine channels
Preza	Insecticide	cyantraniliprole	Modulator of ryanodine receptors
Copper oxychloride	Fungicide	copper oxychloride	Fixation of Cu^2+^ ions in enzymes
Fitotripen	Fungicide	*Trichoderma* sp.	Antagonism and mycoparasitism
Mesurol	Insecticide	methiocarb	Acetylcholinesterase inhibitor
Topas	Fungicide	penconazole	Inhibition of sterol synthesis
Athrin Brio	Insecticide	λ-cyhalothrin	Sodium channel modulator
Plaguicontrol	Insecticide	garlic and chili extracts	Unknown

^a^ Mechanisms of action according to the insecticide resistance action committee (IRAC) mode classification [21,22].

**Table 2:** Annotated metabolites by LC-MS in the alkaloidal extract of *Z. schreberi*.

No ^a^	t_R_ ^b^	[M+H]^+^ (*m*/*z*)	am ^c^ (*m*/*z*)	Error ^d^	Annotation ^e^
**1**	16.7	342.1	342.1718	−3.71	tetrahydrocolumbamine
**2**	22.2	354.2	354.1721	−4.43	*N*-methylcanadine
**3**	25.3	322.1	322.1088	−2.70	berberrubine
**4**	25.9	348.1	348.1225	3.10	chelerythrine
**5**	28.1	334.1	334.1066	3.98	fagaridine
**6**	29.3	336.1	336.1247	−3.33	berberine
**7**	34.2	230.1	230.0809	3.56	γ-fagarine
**8**	38.8	386.2	386.1981	−3.50	muramine
**9**	40.6	320.1	320.0933	−3.19	zanthoxyline
**10**	41.4	350.2	350.1383	2.66	dihydrochelerythrine
**11**	44.1	334.1	334.1071	2.48	norchelerythrine

^a^ Compound numbering **1**–**11** according to the respective total ion chromatogram (TIC) (Figure 4); ^b^ t_R_ = retention time (min); ^c^ Accurate mass obtained by high-resolution mass spectrometry (HRMS) measurements; ^d^ Relative error (in ppm) between accurate mass and theoretical monoisotopic mass of the quasimolecular ion ([M+H]^+^); ^e^ Annotated alkaloids at level 3 according to the confidence levels to communicate metabolite identity by HRMS [26].

**Table 3:** Enzyme activity in protein homogenates of untreated and *Z. schreberi* extract-treated *T. uritcae* adult females.

	Enzymes ^a^			
	GST ^b^	PMO ^c^	Est ^b^	AChE ^b^
Untreated mites	2118 ± 56 ^A^	463 ± 34 ^B^	438 ± 26 ^A^	1257 ± 48 ^A^
*Z. schreberi-*treated mites	235 ± 13 ^B^	1345 ± 41 ^A^	445 ± 31 ^A^	578 ± 41 ^B^

^a^ Test enzymes: AChE = acetylcholinesterase, GST = glutathione *S*-transferase, PMO = P450 monooxygenase; Est = general esterases; ^b^ specific enzyme activity expressed as µmoles of substrate/min/mg protein; ^c^ specific enzyme activity expressed as µmoles of cytochrome P450 equivalent units (CPEU)/min/mg protein. Data expressed as means ± standard deviation (*n* = 9). According to the Tukey test, different uppercase superscript letters indicate significant differences per enzyme.

**Table 4:** Consensus docking strategy employing exponential consensus ranking from docking scores derived from three programs.

*Tu*AChE ^a^						*Tu*GST ^a^						*Tu*PMO ^c^					
No ^b^	V ^c^	M ^c^	G ^c^	ES ^d^	ECR ^e^	No ^b^	V ^c^	M ^c^	G ^c^	ES ^d^	ECR ^e^	No ^b^	V ^c^	M ^c^	G ^c^	ES ^d^	ECR ^e^
**12 ^f^**	−8.70	−121.7	70.2	0.239	1	**13 ^g^**	−8.60	−138.2	52.7	0.233	1	**14 ^h^**	−8.80	−120.9	71.8	0.263	1
**6**	−6.90	−124.8	72.4	0.239	2	**6**	−8.70	−100.3	50.4	0.230	2	**7**	−3.30	−89.7	54.4	0.254	2
**9**	−7.10	−122.4	63.9	0.209	3	**2**	−8.60	−106.4	49.2	0.223	3	**9**	−1.50	−32.4	42.7	0.189	3
**4**	−7.20	−113.1	68.8	0.201	4	**3**	−8.90	−99.2	48.9	0.218	4	**5**	−1.40	−22.7	43.5	0.179	4
**2**	−4.30	−126.7	45.3	0.173	5	**1**	−7.20	−98.2	54.4	0.184	5	**11**	−1.20	−6.5	50.4	0.174	5
**5**	−4.10	−115.3	66.6	0.163	6	**11**	−8.50	−96.7	48.4	0.159	6	**3**	−1.30	−10.1	48.8	0.171	6
**8**	−5.90	−119.5	39.4	0.151	7	**9**	−8.60	−85.5	43.6	0.137	7	**4**	−1.10	−16.7	46.5	0.149	7
**3**	−4.10	−114.7	52.9	0.144	8	**10**	−8.10	−95.4	44.6	0.136	8	**10**	−1.10	−17.5	42.2	0.147	8
**7**	−5.60	−102.2	51.2	0.130	9	**4**	−7.90	−92.3	46.5	0.131	9	**2**	−0.90	3.0	49.2	0.141	9
**1**	−3.10	−103.6	54.8	0.121	10	**5**	−8.00	−88.4	44.8	0.126	10	**8**	−0.50	57.9	47.7	0.115	10
**11**	−3.10	−110.6	50.8	0.111	11	**8**	−7.30	−85.5	48.8	0.125	11	**6**	−1.00	3.1	9.0	0.111	11
**10**	−3.60	−109.6	49.5	0.110	12	**7**	−6.50	−73.6	37.0	0.090	12	**1**	−0.90	5.1	2.4	0.100	12

^a^ Test enzymes: AChE = acetylcholinesterase, GST = glutathione *S*-transferase, PMO = P450 monooxygenase homologically modeled from the respective *T. urticae* enzyme sequences (i.e., D8V7J9, T1K0V7, T1L3S2, and T1L3S2 UniProt entries, respectively); ^b^ Selected ligands docked within the active site of the test enzymes: numbering according to the annotation of Table 2. ^c^ Scores obtained after molecular docking simulations with programs having different search algorithms and scoring functions: V = Vina scores, M = scores MolDock scores, G = GOLD scores; ^d^ ES = exponential scores obtained from docking scores per program through the metrics previously reported [33]. ^e^ ECR = exponential consensus ranking organized from ES. Compounds **12**–**14** are related to the co-crystalized inhibitors retrieved from the respective enzyme templates (PDB ID: 7D9O, 6GSV, and 6C94, respectively), such as ^f^ fluorodonepezil, ^g^ ʟ-γ-glutamyl-S-[(9*S*,10*S*)-10-hydroxy-9,10-dihydrophenanthren-9-yl]-ʟ-cysteinylglycine, and ^h^ *N*-(4-butyl-2-methylphenyl)-*N*′-hydroxyimidoformamide. Background color per column is related to each docking program′s score variation along test compounds **1**–**12** according to a heat map scale: green = worst score, red = best score.

**Table 5:** Enzyme inhibition of *Z. schreberi* extract and isolated alkaloids.

	Enzymes ^a^			
Subs. ^b^	GST	PMO	Est	AChE
extract	8.65 (8.44–8.78)	125 (119–130)	>1000	15.8 (15.1–16.7)
**3**	5.61 (5.49–5.77)	62.7 (60.3–64.8)	>300	12.4 (11.8–13.5)
**4**	11.71 (11.53–11.89)	89.6 (88.8–90.8)	>300	1.86 (1.69–1.98)
**5**	112 (99.3–121)	20.9 (20.1–22.3)	>300	14.6 (13.9–15.7)
**6**	1.24 (1.05–1.45)	386 (371–399)	>300	0.325 (0.306–0.351)
**9**	21.5 (20.9–22.3)	8.37 (8.23–8.49)	289 (263–299)	3.53 (3.21–3.76)
donepezil	-	-	-	0.0135 (0.0118–0.0154)

^a^ Test enzymes: AChE = acetylcholinesterase, GST = glutathione *S*-transferase, PMO = P450 monooxygenase; Est = general esterases; ^b^ Test substances for enzyme inhibition. Data expressed as a half-maximal inhibitory concentration, IC_50_ (expressed as µg/mL for extract and µM for pure compounds), and 95% interval confidence in parenthesis.

## Data Availability

The data presented in this study are available upon request from the corresponding author.
